# Linagliptin Ameliorates Methylglyoxal-Induced Peritoneal Fibrosis in Mice

**DOI:** 10.1371/journal.pone.0160993

**Published:** 2016-08-11

**Authors:** Takuo Nagai, Shigehiro Doi, Ayumu Nakashima, Taisuke Irifuku, Kensuke Sasaki, Toshinori Ueno, Takao Masaki

**Affiliations:** Department of Nephrology, Hiroshima University Hospital, Hiroshima, Japan; IIT Research Institute, UNITED STATES

## Abstract

Recent studies have reported increases of methylglyoxal (MGO) in peritoneal dialysis patients, and that MGO-mediated inflammation plays an important role in the development of peritoneal fibrosis through production of transforming growth factor-β1 (TGF-β1). Linagliptin, a dipeptidyl peptidase-4 inhibitor, exerts anti-inflammatory effects independent of blood glucose levels. In this study, we examined whether linagliptin suppresses MGO-induced peritoneal fibrosis in mice. Male C57/BL6 mice were divided into three groups: control, MGO injection plus saline, and MGO injection plus linagliptin (n = 6 per group). Peritoneal fibrosis was induced by daily intraperitoneal injection of saline containing 40 mmol/L MGO for 21 days. Saline was administered intraperitoneally to the control group. Linagliptin (10 mg/kg) or saline were administrated by once-daily oral gavage from 3 weeks before starting MGO injections. Immunohistochemical staining revealed that linagliptin suppressed expression of α-smooth muscle actin and fibroblast-specific protein-1, deposition of type I and III collagen, and macrophage (F4/80) infiltration. Peritoneal equilibration testing showed improved peritoneal functions in mice treated with linagliptin. Peritoneal injection of MGO increased plasma levels of glucagon-like peptide-1 (GLP-1) in mice, and a further increase was observed in linagliptin-treated mice. Although MGO increased plasma glucose levels, linagliptin did not decrease plasma glucose levels. Moreover, linagliptin reduced the TGF-β1 concentration in the peritoneal fluid of MGO-treated mice. GLP-1 receptor (GLP-1R) was expressed in monocytes/macrophages and linagliptin suppressed GLP-1R expression in MGO-injected mice. These results suggest that oral administration of linagliptin ameliorates MGO-induced peritoneal fibrosis.

## Introduction

Peritoneal dialysis (PD) is a well-established renal replacement therapy for patients with end-stage kidney disease. However, long term exposure to PD fluid leads to inflammation and eventual peritoneal fibrosis that is clinically presented as the failure of peritoneal ultrafiltration [1–5]. Pathologically, in addition to infiltration of inflammatory cells, peritoneal fibrosis is characterized by loss of mesothelial cells, abnormal proliferation of α-smooth muscle actin (α-SMA)-positive myofibroblasts, and significant accumulation of extracellular matrix (ECM) proteins [6–11]. As a major contributor to fibrosis, transforming growth factor-β1 (TGF-β1) from macrophages has been identified in the peritoneum as well as various other tissues [7,12–14]. However, inhibition of TGF-β1 production has not yet been achieved in a clinical setting.

Methylglyoxal (MGO) is a major precursor of advanced glycation end products [15,16] and reportedly increases with elevated levels of blood glucose [17]. Previous studies have revealed that MGO induces inflammation, thus contributing to the progression of diabetic complications [18–21]. To enable ultrafiltration, high concentrations of glucose are used as a hyperosmotic agent in PD fluid, and MGO levels in peritoneal fluid are therefore increased in PD patients [22]. In addition to high glucose in PD fluid, MGO-induced inflammation has recently been reported to participate in the development of peritoneal fibrosis [23]. Furthermore, injection of MGO into the peritoneal cavity has been established as an experimental mouse model of peritoneal fibrosis that is accompanied by infiltration of inflammatory cells [24].

Dipeptidyl peptidase-4 (DPP-4) inhibitors are clinically available and generally well tolerated in type 2 diabetic patients [25]. Previous studies have demonstrated that DPP-4 inhibitors not only improve hyperglycemia, but also protect against tissue damage through various stimuli, such as myocardial fibrosis in db/db−/− mice, renal fibrosis in 5/6 nephrectomy rats, renal ischemia-reperfusion injury, cisplatin nephropathy, atherosclerosis in apolipoprotein E-null mice, and non-alcoholic steatohepatitis and renal fibrosis in streptozotocin-induced diabetic mice [26–31]. Notably, DPP-4 inhibitors increase the circulating levels of intact bioactive glucagon-like peptide (GLP-1), resulting in reduced inflammation and TGF-β1 expression in a non-diabetic rat model [27]. These findings led us to the hypothesis that the DPP-4 inhibitor linagliptin may suppress MGO-induced peritoneal fibrosis.

In this study, we demonstrated that linagliptin suppressed the expression of fibrotic markers, such as α-SMA, fibroblast-specific protein-1 (FSP-1), and type I and III collagen, and inhibited macrophage infiltration in a mouse model of MGO-induced peritoneal fibrosis. We showed that linagliptin improved peritoneal functions by a peritoneal equilibration test (PET) and reduced the TGF-β1 concentration in PD fluid of MGO-treated mice. We also showed that linagliptin increased plasma levels of GLP-1 in MGO-treated mice, even without a significant difference in plasma glucose levels. *In vitro* experiments showed that GLP-1 receptor (GLP-1R) was expressed in the monocyte/macrophage cell line THP-1, but not in human peritoneal mesothelial cells (HPMCs). Lastly, expression of GLP-1R was up-regulated in MGO-injected mice, but it was suppressed by oral administration of linagliptin.

## Materials and Methods

### Animals

Male C57/BL6 mice (10 weeks of age and weighing approximately 25 g) were purchased from Charles River Laboratories Japan (Yokohama, Japan). The animals had free access to laboratory chow and tap water, and were housed in a light- and temperature-controlled room in the Laboratory Animal Center of Hiroshima University (Hiroshima, Japan). The mice were divided into three groups (n = 6 per group). The control group received only saline (100 mL/kg) by intraperitoneal injection for 15 days, 5 consecutive days per week for a total of 3 weeks; the MGO + saline group received intraperitoneal injection of MGO (40 mM) according to the same schedule as the control group plus oral gavage of saline once daily; the MGO + linagliptin group received intraperitoneal injection of MGO plus oral gavage of linagliptin (10 mg/kg) once daily. In all groups, administration of saline or linagliptin was started at 3 weeks before MGO injection. Following 3 weeks of MGO treatment, blood samples and peritoneal tissue were collected under sedation with sodium pentobarbital anesthesia. Measurement of plasma glucose levels was outsourced to SRL (Hiroshima, Japan).

The experimental protocol was approved by the Animal Care and Use Committee at Hiroshima University (permit number: A13-76). All animal experiments were performed in accordance with the National Institutes of Health Guidelines on the Use of Laboratory Animals.

### Histological analysis and immunochemistry

Histology and immunohistochemical staining of 4-μm-thick tissue sections were performed as described previously [32,33] using the following primary antibodies: mouse monoclonal anti-α-SMA antibody (Sigma-Aldrich, St. Louis, MO, USA); rabbit polyclonal anti-FSP-1 antibody (Abcam, Cambridge, UK); rabbit polyclonal F4/80 antibody (Abcam); rabbit polyclonal anti-type I collagen antibody (Abcam); rabbit polyclonal anti-type III collagen antibody (Chemicon International, Temecula, CA, USA); rabbit polyclonal anti-TGF-β1 antibody (Sigma-Aldrich); rabbit polyclonal anti-GLP-1R antibody (Bioss, Woburn, MA, USA).

Type I and III collagen-stained areas were assessed in predetermined fields (×200 magnification) of the submesothelial compact zone, captured by a digital camera, and analyzed using ImageJ software (version 1.44p; National Institutes of Health, Bethesda, MD, USA). The numbers of cells positive for α-SMA, FSP-1, TGF-β1, F4/80, and GLP-1R in the submesothelial compact zone were counted in nine fields at ×200 magnification.

### Peritoneal equilibrium test (PET)

A PET was performed on day 21 after starting MGO injections. Mice were instilled with a 4.25% Dianeal solution (Baxter Health Care, Deerfield, IL, USA) at 100 mL/kg body weight before sacrifice. After 15 minutes, the peritoneal fluid was removed and blood samples were obtained by cardiac puncture. Glucose levels were measured at SRL. The ratio of glucose in drained dialysate obtained at 15 minutes after injection to that obtained immediately after injection was defined as the D/D0 glucose level.

### Enzyme-linked immunosorbent assays (ELISAs) for detection of plasma GLP-1 and TGF-β1 levels in peritoneal fluid

For GLP-1 measurement, blood samples were collected into ethylenediaminetetraacetic acid-coated collection tubes containing a protease inhibitor cocktail and DPP-4 inhibitor, and applied to a GLP-1 protein assay with a GLP-1 ELISA kit (Immuno-Biological Laboratories, Fujioka, Japan).

Peritoneal fluid samples were collected at the time of PET. Following treatment with 1 N HCl and 1.2 N NaOH, they were assessed for TGF-β1 concentrations using a TGF-β1 ELISA kit (R&D Systems, Minneapolis, MN, USA) according to the manufacturer’s instructions.

### Cell culture

HPMCs were isolated from human omentum as described previously. [34] Harvesting of the omentum was permitted by the Medical Ethics Committee of Hiroshima Graduate School of Biomedical Science. Informed consent was obtained from each patient. HPMCs were maintained in M199 medium (Life Technologies, NY, USA) containing 10% fetal bovine serum (FBS) (Nichirei Bio Science, Tokyo, Japan) and penicillin/streptomycin (Nacalai Tesque, Kyoto, Japan).

THP-1 cells were obtained from the American Type Culture Collection (Manassas, VA, USA). The cells were maintained in M199 medium containing 10% FBS and penicillin/streptomycin, and differentiated into macrophages by treatment with 5 ng/mL phorbol 12-myristate 13-acetate (Sigma-Aldrich) for 48 h.

### Western blotting

Western blot analysis was performed as described previously. [35] Primary antibodies used in this study were anti-GLP-1R (Bioss) and anti-glyceraldehyde 3-phosphate dehydrogenase (GAPDH; Sigma-Aldrich). The intensity of each band was determined using ImageJ software.

### Statistical analysis

Results are expressed as means ± standard deviation. Statistical analyses were performed using the Student’s *t-*test or analysis of variance (ANOVA) followed by Tukey’s *post-hoc* test. The Student’s *t*-test was used for analyses of two groups, and ANOVA followed by Tukey’s test for analyses of more than two groups. Values of *P* < 0.05 were considered as statistically significant.

## Results

### Linagliptin suppresses peritoneal cell density and thickening induced by MGO

**Fig 1:**
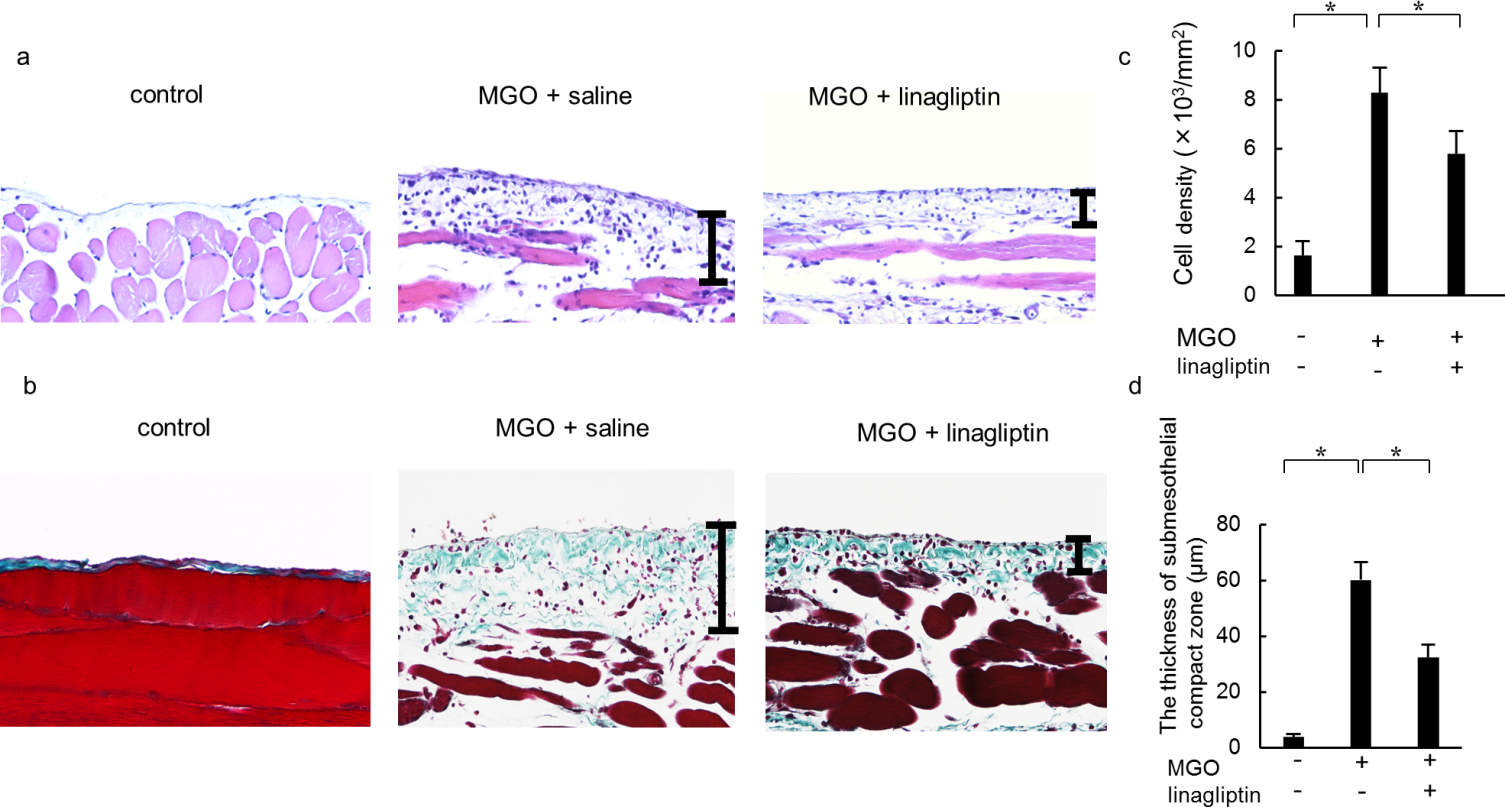
Linagliptin suppresses peritoneal cell density and thickening in methylglyoxal (MGO)-injected mice. (a, b) Representative light microscopy of peritoneal tissues on day 21 (a: hematoxylin-eosin staining; b: Masson’s trichrome staining; original magnification: ×200) in control mice, MGO-injected mice treated with saline, and MGO-injected mice treated with linagliptin. (c, d) The thickness of the submesothelial compact zone increased along with its cellularity until day 21 in mice treated with saline, whereas cell density and zone thickening were suppressed in mice treated with linagliptin. Bars indicate the compact zone area. Statistical analyses were performed using analysis of variance (ANOVA) followed by Tukey’s *post-hoc* test. *P < 0.05.

To evaluate the effect of linagliptin on cell density and thickening in MGO-induced peritoneal fibrosis, we performed hematoxylin-eosin staining (Fig 1A) and Masson’s trichrome staining (Fig 1B). The peritoneal cell density was suppressed in the MGO + linagliptin group compared with the MGO + saline group (Fig 1C). Compared with the control group, the peritoneal tissue of the MGO + saline group showed significant thickening of the submesothelial compact zone. The thickness of the submesothelial compact zone in the MGO + linagliptin group was significantly less than that in the MGO + saline group (Fig 1D).

### Linagliptin decreases the expression of α-SMA and FSP-1 in mice with MGO-induced peritoneal fibrosis

**Fig 2:**
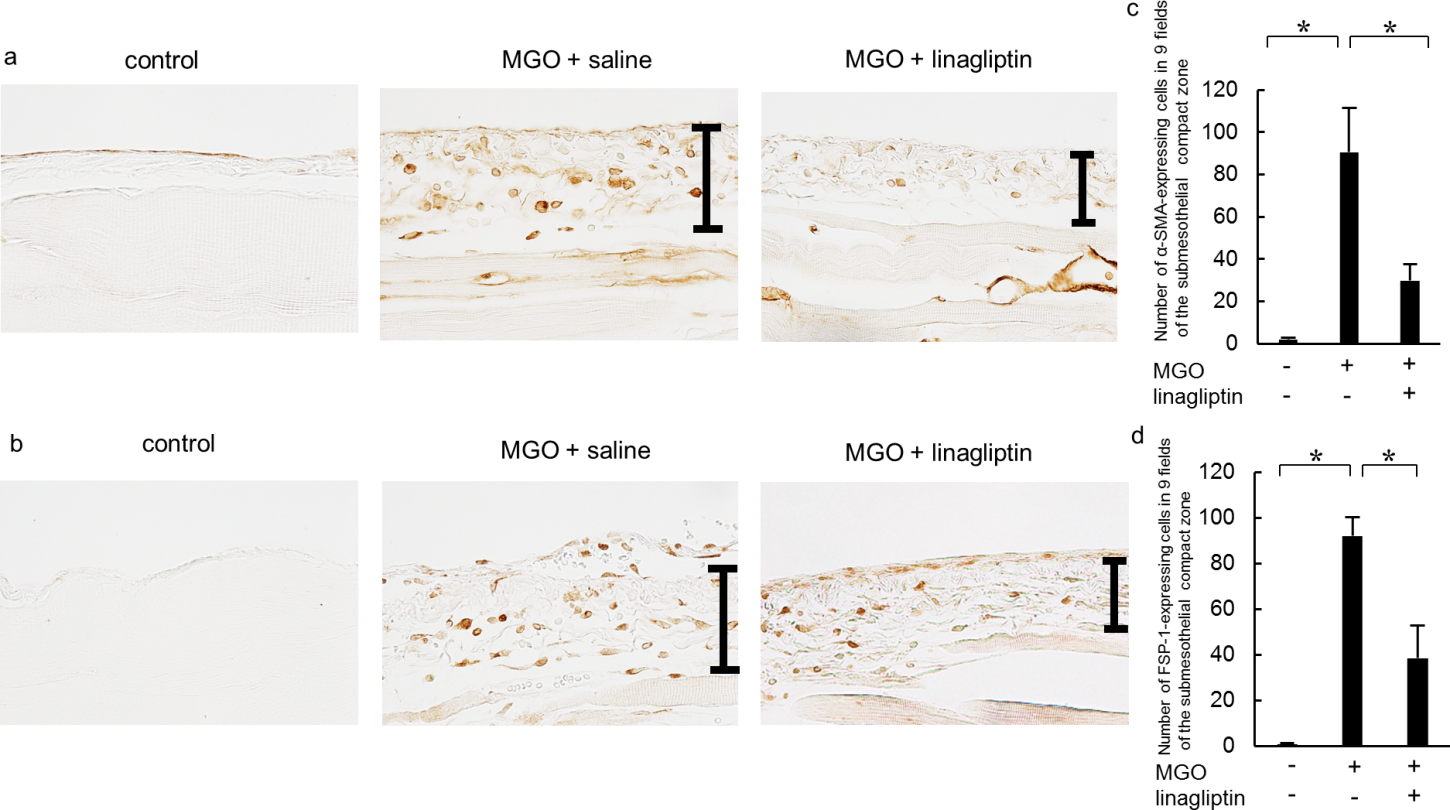
Linagliptin inhibits α-smooth muscle actin (α-SMA) and fibroblast-specific protein-1 (FSP-1) expression in mice with peritoneal fibrosis. Immunohistochemical analyses of (a) α-SMA and (b) FSP-1 expression in peritoneal tissues on day 21 (original magnification: ×200) in control mice, MGO-injected mice treated with saline, and MGO-injected mice treated with linagliptin. (c, d) Accumulation of α-SMA+ and FSP-1+ cells was observed on day 21 in MGO-injected mice treated with saline, whereas the numbers of α-SMA-and FSP-1-expressing cells were significantly lower in MGO-injected mice treated with linagliptin. Statistical analyses were performed using ANOVA followed by Tukey’s *post-hoc* test. *P < 0.05.

We examined the peritoneal expression of α-SMA and FSP-1 as mesenchymal markers. After injection of MGO, an accumulation of α-SMA expression was found in the upper layer of the submesothelial compact zone and in vascular smooth muscle cells (Fig 2A). Linagliptin administration significantly reduced the α-SMA-positive area compared with that in the MGO + saline group (Fig 2C), and also reduced the number of FSP-1-positive cells that had accumulated in the upper layer of the submesothelial compact zone (Fig 2B). Linagliptin administration also significantly reduced the number of FSP-1-positive cells compared with the MGO + saline group (Fig 2D).

### Linagliptin suppresses the expression of type I and III collagen

**Fig 3:**
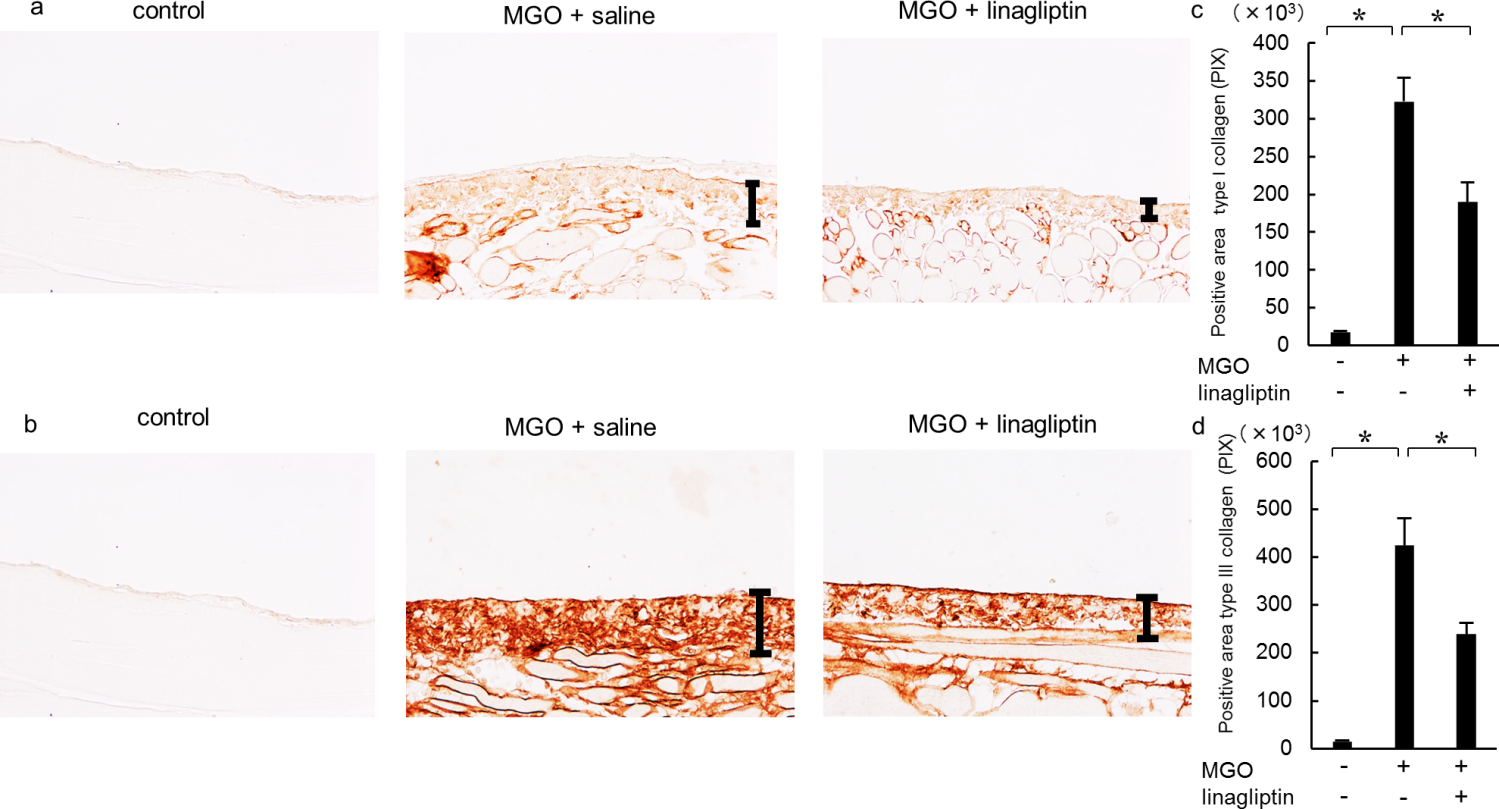
Linagliptin reduces type I and III collagen expression in mice with peritoneal fibrosis. Immunohistochemical analyses of (a) type I collagen and (b) type III collagen expression in peritoneal tissues on day 21 (original magnification: ×200) in control mice, MGO-injected mice treated with saline, and MGO-injected mice treated with linagliptin. The numbers of type I and III collagen pixels were increased on day 21 in MGO-injected mice treated with saline but significantly decreased in MGO-injected mice treated with linagliptin (c, d). Statistical analyses were performed using ANOVA followed by Tukey’s *post-hoc* test. *P < 0.05.

Type I and III collagens are ECM proteins that were expressed in the submesothelial compact zone (Fig 3A and 3B). In the MGO + saline group, type I and III collagens were diffusely expressed, and the positive areas were significantly larger than those in the control group. Linagliptin administration significantly reduced the expression of type I and III collagens compared with that in the MGO + saline group (Fig 3C and 3D).

### Linagliptin ameliorates infiltration of monocytes/macrophages and TGF-β1 expression

**Fig 4:**
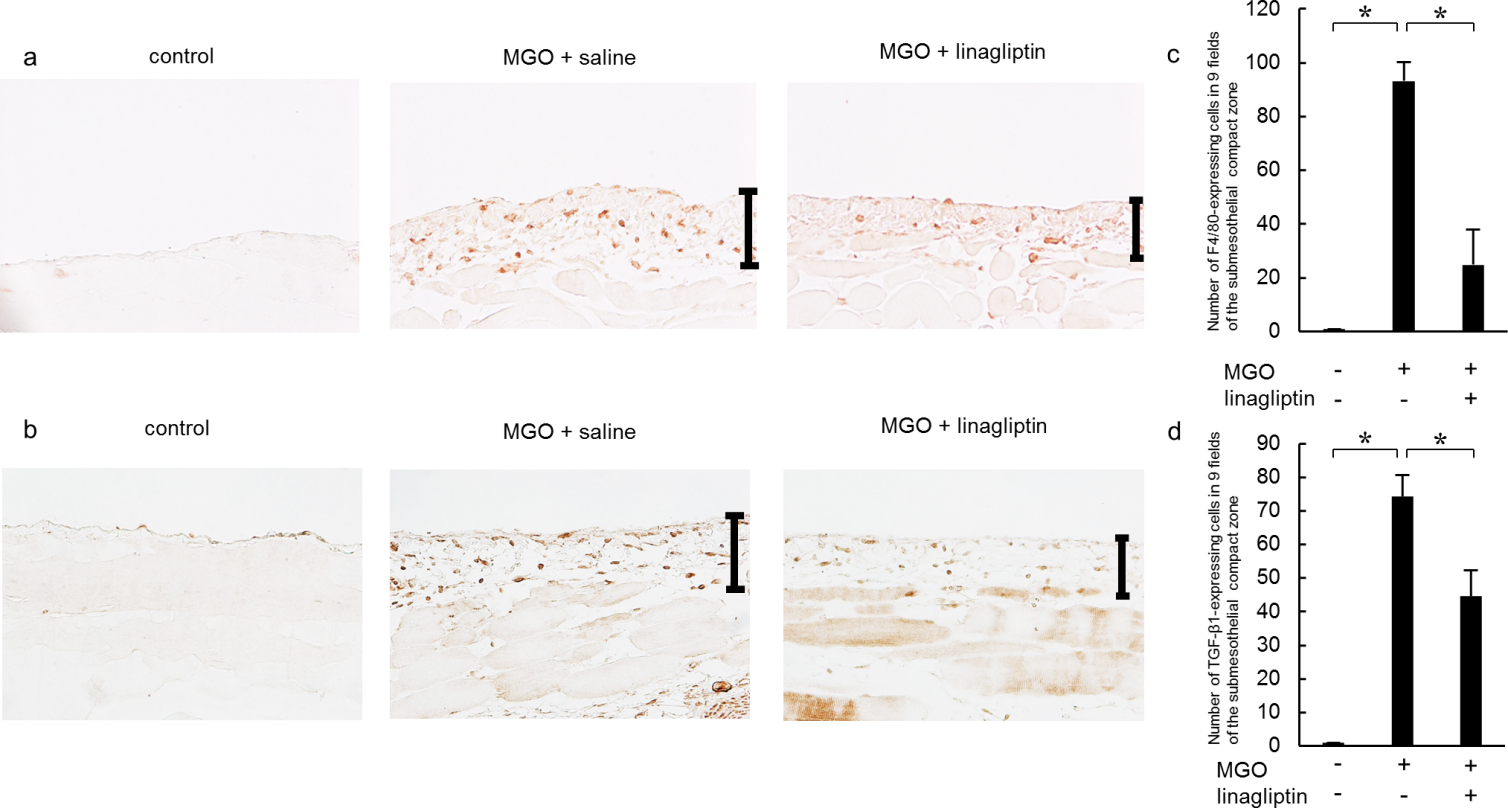
Linagliptin attenuates monocyte/macrophage infiltration and transforming growth factor-β1 (TGF-β1) expression in mice with peritoneal fibrosis. (a) Immunohistochemical analysis of F4/80 expression in peritoneal tissue on day 21 in control mice, MGO-injected mice treated with saline, and MGO-injected mice treated with linagliptin. (b) Immunohistochemical analysis of TGF-β1 expression in peritoneal tissues on day 21 in MGO-injected mice treated with saline and MGO-injected mice treated with linagliptin. (c) The number of F4/80-expressing cells increased until day 21 in MGO-injected mice treated with saline, but significantly decreased by day 21 in MGO-injected mice treated with linagliptin. (d) The number of TGF-β1-expressing cells increased until day 21 in MGO-injected mice treated with saline, but significantly decreased by day 21 in MGO-injected mice treated with linagliptin. Statistical analyses were performed using ANOVA followed by Tukey’s *post-hoc* test. *P < 0.05 (original magnification in a and b: ×400).

To investigate monocyte/macrophage infiltration into the peritoneum, we stained the tissue sections with an anti-F4/80 antibody. In the MGO + saline group, we observed numerous monocytes/macrophages in the submesothelial zone (Fig 4A). Linagliptin administration significantly inhibited monocyte/macrophage infiltration compared with that in the MGO + saline group (Fig 4C).

TGF-β1 is a major contributor to the development of fibrosis. Therefore, we evaluated the numbers of TGF-β1-expressing cells. In the MGO + saline group, the number of TGF-β1-expressing cells was higher than that in the control group (Fig 4B). Linagliptin administration significantly reduced the number of TGF-β1-expressing cells compared with that in the MGO + saline group (Fig 4D).

### Linagliptin increases plasma GLP-1 levels and improves peritoneal functions and TGF-β1 levels in peritoneal fluid without reducing plasma glucose levels

**Fig 5:**
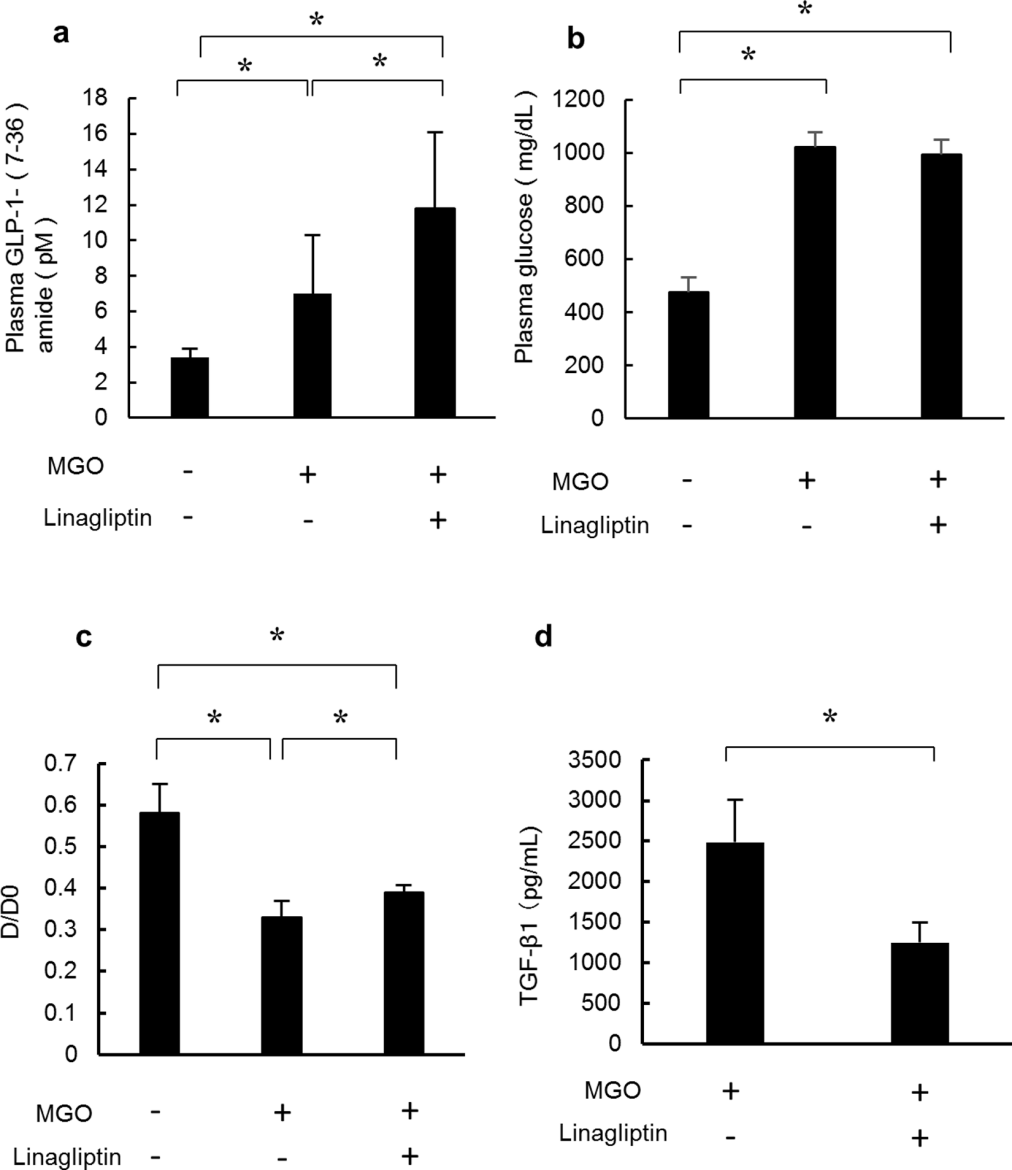
Linagliptin increases plasma GLP-1 (7–36) amide, improves peritoneal function, and decreases TGF-β1 levels in peritoneal fluid. (a) Plasma GLP-1 (7–36) amide levels were analyzed in control mice, MGO-injected mice treated with saline, and MGO-injected mice treated with linagliptin. The plasma GLP-1 (7–36) amide level was significantly higher in MGO-injected mice treated with linagliptin than in MGO-injected mice treated with saline or in control mice. (b) Plasma glucose levels were measured in control mice, MGO-injected mice treated with saline, and MGO-injected mice treated with linagliptin. The plasma glucose level was significantly higher in MGO-injected mice treated with or without linagliptin than in control mice. Plasma glucose levels did not differ in MGO-injected mice treated with linagliptin or saline. (c) Peritoneal absorption of glucose from the dialysate (D/D0) was assessed in the three treatment groups during a 15-minute instillation of dialysate (4.25% Dianeal) at 100 mL/kg body weight. D/D0 values were significantly lower in MGO-injected mice treated with saline than in control mice, but were significantly increased in MGO-injected mice treated with linagliptin. (d) TGF-β1 levels in peritoneal fluid were assessed by ELISA and found to be significantly lower in MGO-injected mice treated with linagliptin than in MGO-injected mice treated with saline. Statistical analyses were performed using ANOVA followed by Tukey’s *post-hoc* test (a–c) or the Student’s *t*-test (d). *P < 0.05.

DPP-4 inhibitors are known to up-regulate plasma GLP-1 levels, resulting in lowered plasma glucose levels through secretion of insulin. Therefore, we investigated the effect of linagliptin on plasma GLP-1 and glucose levels in MGO-injected mice. Oral administration of linagliptin increased plasma GLP-1 levels in the MGO + linagliptin group (Fig 5A), but it did not decrease plasma glucose levels (Fig 5B). A PET was also performed on day 21 after starting MGO injections to assess functional alterations of the peritoneal membrane. The dialysate obtained during the PET represented D/D0 glucose. D/D0 glucose levels were significantly higher in the MGO + linagliptin group than in the MGO + saline group (Fig 5C). Considering the role of TGF-β1 in the development of fibrosis, we investigated TGF-β1 levels in peritoneal fluid. The TGF-β1 levels were significantly lower in the MGO + linagliptin group than in the MGO + saline group (Fig 5D).

### GLP-1R is expressed in monocytes/macrophages and up-regulation of MGO-induced GLP-1R is suppressed by linagliptin

**Fig 6:**
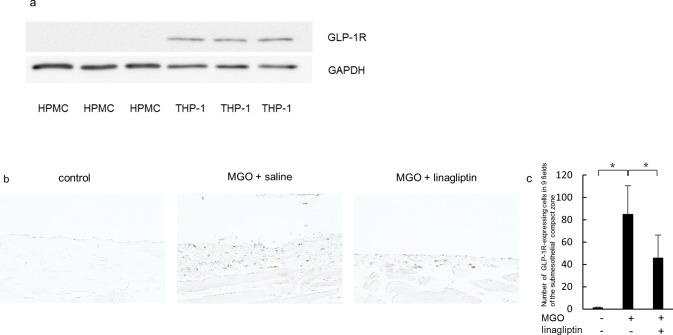
GLP-1R is expressed in infiltrated inflammatory cells and MGO-induced GLP-1 expression is suppressed by linagliptin. (a) Expression of GLP-1R was examined by western blotting. GLP-1R was expressed in the monocyte/macrophage cell line THP-1, but not in HPMCs. (b) Immunohistochemical analysis of GLP-1R expression in peritoneal tissue on day 21 in control mice, MGO-injected mice treated with saline, and MGO-injected mice treated with linagliptin. (c) The number of GLP-1R-expressing cells was increased until day 21 in MGO-injected mice treated with saline, but it was significantly decreased by day 21 in MGO-injected mice treated with linagliptin. Statistical analyses were performed using ANOVA followed by Tukey’s *post-hoc* test. *P < 0.05. (original magnification in b: ×200).

To investigate GLP-1R expression in THP-1 cells and HPMCs, we performed western blot analysis of GLP-1R and GAPDH. Expression of GLP-1R was observed in THP-1 cells, but not in HPMCs (Fig 6A).

We also investigated the expression level of GLP-1R in MGO-injected mice treated with or without linagliptin. GLP-1 expression was up-regulated in MGO-injection mice, whereas its expression was suppressed by linagliptin treatment (Fig 6B and 6C).

## Discussion

The present study has demonstrated that linagliptin ameliorates MGO-induced peritoneal fibrosis in mice. This protective effect is accompanied by a reduction in the infiltration of inflammatory cells into the peritoneal membrane. Linagliptin increases plasma GLP-1 levels without a change in the serum glucose level, but it decreases TGF-β1 levels in PD fluid. GLP-1R is expressed in THP-1 cells, but not in HPMCs, suggesting that the beneficial effect of linagliptin is exerted through the GLP-1-mediated reduction of inflammation. Furthermore, we found that the increased expression of GLP-1R in MGO-injected mice is suppressed by linagliptin treatment. Because MGO-induced inflammation plays an important role in the development of peritoneal fibrosis through the production of TGF-β1, the protective effects of linagliptin may be mediated by the attenuation of inflammation. Importantly, linagliptin is an available anti-diabetic drug, and its safety has been established in the clinical setting. Therefore, inhibition of DPP-4 activity may be an attractive therapy for patients undergoing PD.

Lenski et al. reported that the DPP-4 inhibitor sitagliptin prevents myocardial fibrosis in 10-week-old db/db−/− mice [26]. In addition, Kanasaki et al. showed that linagliptin ameliorates kidney fibrosis in streptozotocin-induced diabetic mice [31]. These findings suggest that linagliptin suppresses fibrosis in experimental animal models of diabetes. Notably, our data demonstrate an anti-fibrotic effect of linagliptin without a change in serum glucose levels. Therefore, the beneficial effects of linagliptin on tissue fibrosis may be independent of decreased plasma glucose levels in animal models of diabetes.

We show that linagliptin suppresses both inflammation and peritoneal fibrosis. In the clinical setting, long-term exposure to PD fluid can cause chronic inflammation, an important contributor to the progression of peritoneal fibrosis. Previous studies have reported that a DPP-4 inhibitor prevents exacerbation of diabetic nephropathy through anti-inflammatory effects in a rat model of type 1 diabetes [36]. In addition, a DPP-4 inhibitor reportedly suppresses nuclear factor-kB activity in the kidney [36]. In this study, we could not demonstrate an inhibitory effect of linagliptin on TGF-β1-induced α-SMA expression in HPMCs (data not shown). These findings suggest that linagliptin suppresses peritoneal fibrosis by inhibiting inflammation.

The present data show that administration of linagliptin increases GLP-1 levels. It has been previously reported that a single dose of linagliptin increases basal GLP-1 levels after 24 hours in mice and rats [37]. Moreover, Kodera et al. reported expression of GLP-1R in THP-1 cells and attenuation of glucose-induced *TNF-α* and *IL-1β* gene expression by the GLP-1R agonist exendin-4 [38]. In this study, we also show GLP-1R expression in THP-1 cells, and that linagliptin decreases GLP-1R expression together with a reduction of inflammation. These findings raise the possibility that the anti-inflammatory effects of linagliptin are exerted through increased GLP-1 levels. However, we could not clarify the precise mechanism by which linagliptin and GLP-1 suppress MGO-induced inflammation in THP-1 cells. Further studies are therefore needed to determine the precise mechanism of the anti-fibrotic effects of linagliptin.

MGO is a major precursor in the formation of advanced glycation end products,and contributes to diabetic complications and cancer [39]. A previous study has reported that MGO induces epithelial-mesenchymal transition, leading to peritoneal fibrosis [40]. However, we did not observe MGO-stimulated α-SMA expression in HPMCs (data not shown). Therefore, the fibrotic effect of MGO is likely mediated through TGF-β1 production. In addition, we show that peritoneal injection of MGO induces TGF-β1 expression as well as inflammation, and that linagliptin suppresses both processes. Taken together, it appears that MGO-induced inflammation causes TGF-β1 production, resulting in peritoneal fibrosis.

In summary, we have demonstrated that linagliptin attenuates MGO-induced peritoneal fibrosis in mice and suppresses the infiltration of inflammatory cells into peritoneal tissue as well as *in vivo* TGF-β1 levels in peritoneal fluid. In addition to the histological findings, linagliptin improves peritoneal functions. Although linagliptin increased plasma GLP-1 levels, the plasma glucose level did not differ between untreated and linagliptin-treated mice. Lastly, we show that GLP-1R is expressed in monocytes/macrophages, and that MGO-induced GLP-1R expression is suppressed by linagliptin treatment. These findings suggest that the pharmacological target of linagliptin for treatment of peritoneal fibrosis may be infiltrated inflammatory cells. Considering that linagliptin is a clinically available drug with an established safety profile, its administration may represent a novel strategy to prevent peritoneal fibrosis.
